# Developing an integrated microsimulation model for the impact of fiscal policies on child health in Europe: the example of childhood obesity in Italy

**DOI:** 10.1186/s12916-021-02155-6

**Published:** 2021-11-30

**Authors:** Davide Rasella, Lorenzo Richiardi, Nicolai Brachowicz, H. Xavier Jara, Mark Hanson, Delia Boccia, Matteo G. Richiardi, Costanza Pizzi

**Affiliations:** 1 ISGlobal, Hospital Clínic - Universitat de Barcelona, Carrer Rosselló 132, 08036 Barcelona, Spain; 2 Department of Medical Sciences, University of Turin, Turin, Italy; 3 Centre for Microsimulation and Policy Analysis, Institute for Social and Economic Research, University of Essex, Colchester, UK; 4 Institute of Developmental Sciences and NIHR Biomedical Research Centre, University of Southampton and University Hospital Southampton, Southampton, UK; 5 Faculty of Population and Health Policy, London School of Hygiene and Tropical Medicine, London, UK

**Keywords:** Fiscal policies, Poverty alleviation, Microsimulation, Child health, Child overweight, Child obesity

## Abstract

**Background:**

We developed an integrated model called Microsimulation for Income and Child Health (MICH) that provides a tool for analysing the prospective effects of fiscal policies on childhood health in European countries. The aim of this first MICH study is to evaluate the impact of alternative fiscal policies on childhood overweight and obesity in Italy.

**Methods:**

MICH model is composed of three integrated modules. Firstly, module 1 (M1) simulates the effects of fiscal policies on disposable household income using the tax-benefit microsimulation program EUROMOD fed with the Italian EU-SILC 2010 data. Secondly, module 2 (M2) exploits data provided by the Italian birth cohort called Nascita e Infanzia: gli Effetti dell’Ambiente (NINFEA), translated as Birth and Childhood: the Effects of the Environment study, and runs a series of concatenated regressions in order to estimate the prospective effects of income on child body mass index (BMI) at different ages. Finally, module 3 (M3) uses dynamic microsimulation techniques that combine the population structure and incomes obtained by M1, with regression model specifications and estimated effect sizes provided by M2, projecting BMI distributions according to the simulated policy scenarios.

**Results:**

Both universal benefits, such as universal basic income (BI), and targeted interventions, such as child benefit (CB) for poorer households, have a significant effect on childhood overweight, with a prevalence ratio (PR) in 10-year-old children—in comparison with the baseline fiscal system—of 0.88 (95%CI 0.82–0.93) and 0.89 (95%CI 0.83–0.94), respectively. The impact of the fiscal reforms was even larger for child obesity, reaching a PR of 0.67 (95%CI 0·50–0.83) for the simulated BI and 0.64 (95%CI 0.44–0.84) for CB at the same age. While both types of policies show similar effects, the estimated costs for a 1% prevalence reduction in overweight and obesity with respect to the baseline scenario is much lower with a more focalised benefit policy than with universal ones.

**Conclusions:**

Our results show that fiscal policies can have a strong impact on childhood health conditions. Focalised interventions that increase family income, especially in the most vulnerable populations, can help to prevent child overweight and obesity. Robust microsimulation models to forecast the effects of fiscal policies on health should be considered as one of the instruments to reach the Health in All Policies (HiAP) goals.

**Supplementary Information:**

The online version contains supplementary material available at 10.1186/s12916-021-02155-6.

## Background

Fiscal policies, including fiscal benefits and tax reductions, are interventions that can quickly and effectively change the income of poor households. The literature points to family income, considered as a general indicator of socio-economic position (SEP) [1], as one of the strongest socio-economic determinants of health [2, 3]. Increasing household income, especially among the most vulnerable families, could prevent several health outcomes such as overweight and obesity that represent a serious public health concern in high-income countries and increasingly in low- and middle-income countries [4].

On the one hand, some studies have shown strong negative associations between household income and child obesity [5–7], suggesting that implementing income subsidies, especially among those families that belong to the first deciles of the income distribution, could relax their economic constraints and free resources that may be spent on improving their dietary intake and adopting healthier physical activities. On the other hand, although several mathematical models for obesity reduction have been developed, they all focus on postnatal interventions on diet, physical activity and other lifestyle-related practices, ignoring income and socio-economic position factors [8–10].

The aim of our study is twofold. Firstly, we developed a flexible three-part integrated microsimulation model as a useful policy design tool for investigating the prospective effects of poverty alleviation fiscal policies on child health outcomes, in line with the Health in All Policies (HiAP) framework [11]. Secondly, we provided a case study for Italy that evaluates the potential effects of eight different simulated fiscal policies on overweight and child obesity. The simulated policies range from basic income policies that ensure a universal yearly basic income to more targeted policies such as monthly child benefits to low-income families with children under 5 years of age. These fiscal policies were chosen to allow for identifying potential dose responses. Our aim is to contribute to the decision-making process by offering an integrated approach that permits to evaluate the prospective costs and effects of several fiscal policy interventions on child health.

## Methods

### Study design

The modelling strategy involves two phases and three integrated modules. The first phase comprises two modules. Firstly, we simulate the prospective effects of a variation in benefit policies on equivalised income using EUROMOD (module 1 (M1)). Secondly, we run a series of concatenated regressions to estimate the parameters of interest of several relationships using NINFEA variables (module 2 (M2)). In the second phase, we apply the estimated parameters obtained from M2 to the simulated population obtained from M1 in order to get a simulated BMI distribution (module 3 (M3)).

**Fig. 1:**
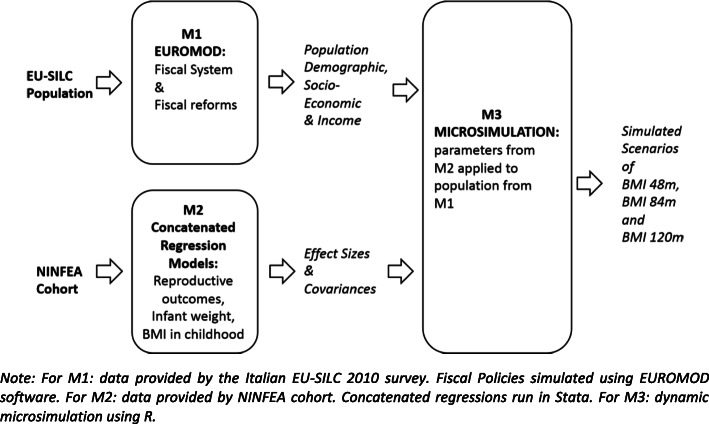
Structure of the MICH model: flow of input, output data, and parameters between M1, M2 and M3 modules

The overall structure of our Microsimulation for Income and Child Health (MICH) model and flow of inputs and outputs for each stage are shown in Fig. 1. A more detailed description of each phase and module is provided in the following paragraphs. Further information on the modelling process and its parameters is provided in Additional file 1 [12–15], in accordance with the international reporting guidelines recommended by the International Society for Pharmacoeconomics and Outcomes Research and the Society for Medical Decision Making (ISPOR-SMDM) [16].

### Data sources

EUROMOD is a tax-benefit microsimulation model for the European Union (EU) and the United Kingdom (UK). It is a software that allows to compute the effects of taxes and benefits on household incomes and work incentives for the population of each country and for the EU as a whole in a standardised and comparable manner [17].

EUROMOD covers the 28 member states and is updated to recent policy systems using data from the European Union Statistics on Income and Living Conditions (EU-SILC) as the input database [18], supported by Directorate-General for Employment, Social Affairs and Inclusion (DG EMPL) of the European Commission [19, 20]. A more detailed description of EUROMOD is provided in Additional file 2 [21].

We use the Italian EU-SILC 2010 data as the input population for EUROMOD, in order to be consistent with the income values of the Italian NINFEA 2011 cohort, given that Italian EU-SILC 2011 data for EUROMOD was not available. The baseline scenario is based on the tax-benefit system corresponding to June 2018, given that this was the most recent system when this study was carried out.

The Nascita e Infanzia: gli Effetti dell’Ambiente (NINFEA, translated as Birth and Childhood: the Effects of the Environment) project [22, 23] is an Italian birth cohort study that aims at investigating the effect of several exposures acting during pre-natal and early post-natal life on later health. Individuals of the cohorts are children whose mothers voluntarily accepted to participate and had enough knowledge of the Italian language to complete the online questionnaires. The first baseline questionnaire on general health and exposures was fulfilled before and during pregnancy. A more detailed description of NINFEA is provided in Additional file 3 [24]. We used the NINFEA database version 02.2019. This database consists of 6625 mothers and 7423 pregnancies. Data on demographic and socio-economic factors of each household were collected using the baseline questionnaire completed during pregnancy. Using these aforementioned variables, namely parental age, cohabitation status, education, country of birth, occupation, house size and type, and family size, and external data from the Italian EU-SILC 2011 survey, the Equivalised Household Income Indicator (EHII) [22] (an indicator of the equivalised total disposable household income at baseline) was constructed for the NINFEA participants.

Children’s birth weight and gestational age at birth were registered at birth. Weight at 6 months of age was ascertained using the 6-month questionnaire, and weight at 18 months of age was obtained from the 18-month questionnaire. From the corresponding follow-up questionnaires at 4, 7 and 10 years of age, our NINFEA dataset includes 4232, 2152 and 973 measurements of children’s weight and height, respectively. These two measurements were used to calculate each children’s body mass index (BMI) at each follow-up. Body mass index (BMI) is computed as the ratio between weight (in kilogrammes) and squared height (in metres). Overweight and obesity for each age were defined according to the official International Obesity Task Force (IOTF) cut-offs [25].

#### M1: EUROMOD and the fiscal reform scenarios

The first module (M1—the tax-benefit microsimulation model) uses EUROMOD capabilities to simulate the prospective effects of eight benefit policies on household disposable income. EUROMOD produces an output dataset that contains a population that is the same as the Italian EU-SILC 2010 sample (46,788 individuals), but with added information on disposable income for each individual, based on the specific, actual or hypothetical policy system considered. This aforementioned output dataset provided individual disposable income data that was aggregated for each household in order to compute firstly the equivalised household size and, later on, the equivalised household income. This adjustment was done in compliance with the modified equivalence scale suggested by the Organisation for Economic Cooperation and Development (OECD) [26]. For reasons of comparability between the Italian EU-SILC 2010 and Italian NINFEA 2011 cohort analyses, we excluded families with more than 7 members or families with no children less than 5 years old.

The simulated fiscal interventions are shown in Table 1. Each pair of simulated policies was implemented with two different levels of intensity regarding the benefit amounts, but keeping the same rules for eligibility and recipients among them, with the aim to evaluate the potential dose-response effects.

**Table 1:** Simulated tax-benefit scenarios

	Baseline (BS)	Basic income (BI)		Poverty reduction (PR)		New-borns benefit (NB)		Child benefit (CB)	
		BI1	BI2	PR1	PR2	NB1	NB2	CB1	CB2
Eligibility	–	All	All	Households with per capita income < €500 monthly	Households with per capita income < €500 monthly	Households with per capita income < €500 monthly	Households with per capita income < €500 monthly	Households with per capita income < €500 monthly	Households with per capita income < €500 monthly
Benefit amount	–	€100	€100	€100	€100	€500	€500	€500	€500
Periodicity	–	Yearly	Monthly	Yearly	Monthly	Yearly	Monthly	Yearly	Monthly
Recipients	–	All household members	All household members	All household members	All household members	Every child < 1 year old	Every child < 1 year old	Every child < 5 years old	Every child < 5 years old

*Note*: Each simulated fiscal policy has two levels of intensity keeping other features fixed. The same benefit amount is given once a year or once a month

Baseline scenario (BS) was simulated applying the actual 2018 Italian fiscal system on the Italian EU-SILC 2010 data. Basic income scenarios, BI1 and BI2, consist of benefit amounts of €100 per year or per month, respectively, to all citizens without eligibility requirements. Poverty reduction scenarios, PR1 and PR2, simulate poverty-relief interventions of €100 per year or per month, respectively, for each member of a household with a per capita disposable income of less than €500 per month. New-borns benefit scenarios, NB1 and NB2, simulate more targeted fiscal interventions of €500 per year or per month, respectively, for each child less than 1 year old in households with an equivalised disposable income of less than €500 per month. Child benefit scenarios, CB1 and CB2, simulate the new-borns benefits scenarios but with the only difference being that the eligibility rule regarding the age threshold for recipients is raised from less than 1 year of age to 5 years of age. Consequently, these CB1- and CB2-simulated policies reach a larger number of households than the new-borns benefit scenarios.

These eight simulated policies with two different intensities allowed to compute marginal benefits, which are a normalised measure of the effectiveness of the different policy instruments, and can be used to compare among the prospective health effects of each alternative.

EUROMOD also provides the overall cost for the public budget and, therefore, after comparison with the baseline, the cost of the changes implemented in the counterfactual scenarios, which can be used to calculate the marginal benefit (health outcome gain compared with the policy cost) of the reforms.

#### M2: the concatenated regression models in the NINFEA birth cohort

The aim of this module is to estimate the regression parameters of interest and the corresponding variance-covariance matrix that are required later in the third module.

The structure of the concatenated regressions models fitted on the NINFEA data is described in the following equations:

1 $$ GA={\alpha}_{ga}+{\delta}_{ga}\mathrm{EHII}+\Sigma\ {\beta}_{ga 5}{\mathrm{X}}_s+\varepsilon $$

2 $$ BW={\alpha}_{bw}+{\beta}_{bw}\mathrm{GA}+{\delta}_{bw}\mathrm{EHII}+\Sigma\ {\beta}_{bw\_s}{\mathrm{X}}_s+\varepsilon $$

3 $$ {WT}_{6m}={\alpha}_{wt6}+{\beta}_{wt6\_ 1} BW+{\beta}_{wt6\_ 2}\mathrm{GA}+{\delta}_{wt6}\mathrm{EHII}+\Sigma\ {\beta}_{wt6\_s}{X}_s+\varepsilon $$

4 $$ {WT}_{18m}={\alpha}_{wt18}+{\beta}_{wt18\_ 1}{WT}_{6m}+{\beta}_{wt18\_ 2} BW+{\delta}_{wt18} EHII+\varSigma\ {\beta}_{wt18\_s}{X}_s+\varepsilon $$

5 $$ {BMI}_{48m}={\alpha}_{b 48}+{\beta}_{b 48\_ 1}{WT}_{18m}+{\beta}_{b 48\_ 2}{WT}_{6m}+{\delta}_{b 48} EHII+\varSigma\ {\beta}_{b 48\_s}{X}_s+\varepsilon $$

6 $$ {BMI}_{84m}={\alpha}_{b 84}+{\beta}_{b 84\_ 1}{BMI}_{48m}+{\beta}_{b 84\_ 2}{WT}_{18m}+{\delta}_{b 84} EHII+\varSigma\ {\beta}_{b 84\_s}{X}_s+\varepsilon $$

7 $$ {BMI}_{120m}={\alpha}_{b 120}+{\beta}_{b 120\_ 1}{BMI}_{84m}+{\beta}_{b 120\_ 2}{BMI}_{48m}+{\delta}_{b 120} EHII+\varSigma\ {\beta}_{b 120\_s}{X}_s+\varepsilon $$

GA and BW stand for gestational age and child’s birth weight, respectively. WT_6m_ and WT_18m_ stand for child’s weight at 6 and 18 months after being born, respectively. BMI_48m_, BMI_84m_ and BMI_120m_ stand for child’s body mass index (BMI) at 4, 7 and 10 years of age, respectively. As described in detail in the paper by Pizzi et al. [22], an indicator of the EU-SILC-based equivalised total disposable household income (the Equivalised Household Income Indicator (EHII)) was constructed for the NINFEA participants within the framework of the H2020 LifeCycle project [27]. In brief, the EHII was constructed using external data provided by the Italian EU-SILC 2011 survey and individual and household characteristics available in the NINFEA cohort, namely parental age, cohabitation status, education, country of birth and occupation, house size and type, and family size. The EHII is the log transformation of the equivalised household disposable income as used in Pizzi et al. [22]. In all equations, EHII is the income indicator (log-transformed), with *δ* being the estimated coefficient of interest for the income indicator.

Moreover, *α* is the intercept—different for each regression—*Σ β*_*s*_ *X*_*s*_ is the sum of sex of child, maternal country of birth and age at delivery. *ε* is the error component. The underlying assumption is that all outcomes analysed are influenced by the two previous ones and by the other factors cited above. For each independent variable, these models provide estimated effect sizes, confidence intervals, and the corresponding variance-covariance matrix required by module 3 (M3).

#### M3: integrating outputs from modules 1 and 2 and creating microsimulation scenarios

The last module of the MICH model applies effect sizes from M2 to the population obtained from M1. The effects of EHII on health outcomes, used to simulate the impact of fiscal policies, were estimated by the *δ* coefficients of the multivariable regressions described above. From M1 output, we select the population of children less than 5 years old and expand it using the Italian EU-SILC 2011 survey sample weights, obtaining a study population of 30,910 children.

Using the same set of concatenated multivariable linear regressions shown above, with the outputs from M1 and M2 used as inputs and the estimated alphas, betas and standard errors of the regressions from M2, the integrated model estimates the distribution of gestational age (GA) for the population of children under 1 year old using regression equation 1. The obtained distribution of gestational age (GA) is successively introduced in regression equation 2 together with the same set of demographic and socio-economic variables, including the equivalised household disposable income from M1. Regression equations from 3 to 7 use the same principle, creating a flow of outputs used as inputs for the next regression model and allowing us to simulate the final body mass index (BMI) distributions at 18, 48 and 84 months, in a sequential order.

For each outcome and each scenario, 1000 simulations were performed using the Monte Carlo sampling method [28]. This allows the main parameter values, in our case the estimated alphas and betas of the regression equations, to vary in each simulation cycle according to their assumed underlying distributions and their variance-covariance matrix. The number of simulations was chosen after verifying that the estimates were stable, and further runs were neither modifying our point estimates, corresponding standard errors, nor other aspects of the simulation.

Because the intercepts were obtained from the regression models applied to the NINFEA cohort, which is not representative of the Italian population, for M2, we needed to calibrate their values. The calibration was achieved varying the alpha of the regression model 1 described above, in order to obtain the lower sum of squared errors (SSE) in comparison with the Italian national prevalence of premature births, and for regression model 2 with the average birth weight, both from the “Certificato di Assistenza al Parto” (CEDAP, translated as Italian birth registry) of the year 2011 [29].

All scenarios were compared in terms of prevalence ratios, using the selected scenario as the numerator and the real fiscal scenario as the denominator. Marginal benefits were obtained by dividing the cost of the fiscal intervention, provided by EUROMOD in M1, by the prevalence difference between scenarios.

M1 was executed in EUROMOD version 3.0.0, and its output processed in STATA version 14. M2 and M3 were coded and implemented in R version 3.6.3.

## Results

Table 2 shows the baseline values corresponding to health outcomes, demographic and socio-economic variables and simulated parameters that were used to build our three-part MICH model.

**Table 2:** Estimated means, percentages and parameters used in the M1, M2 and M3 modules of the MICH model

	M1: EUROMOD (EU-SILC)	M2: NINFEA cohort	M3: Baseline simulated values^a^
Health outcomes			
Gestational age (weeks)	–	39.5 [1.8]	39.6 [0.28]
Birth weight (kg)	–	3237 [499]	3218 [66]
Weight at 6 months (kg)	–	7539 [942]	7774 [241]
Weight at 18 months (kg)	–	11,162 [1285]	11,286 [256]
BMI at 48 months	–	15.6 [1.7]	15.5 [0.2]
BMI at 84 months	–	15.9 [2.1]	16.0 [0.5]
BMI at 120 months	–	17.3 [2.6]	17.9 [1.0]
Demographic and socio-economic predictors			
Female gender	51.60%	49.30%	–
Log of Equivalised income	7.02 [0.67]	7.38 [0.26]	–
Foreign citizenship of the mother	16.80%	4.20%	–
Age of the mother	33.6 [5.2]	33.3 [4.4]	–

*Note*: Weight in kilogrammes. Estimated mean values (with standard deviations in brackets) or percentages

^a^Distribution of the means of the total runs

The mean of log of the household equivalised income at baseline in the Italian EU-SILC 2010 population is lower than in the NINFEA cohort (7.02 and 7.38, respectively). However, the percentage of mothers not born in Italy in the Italian EU-SILC 2010 dataset is higher than in the NINFEA cohort (16.8% and 4.2%, respectively). Modules 1 and 2 portrait similar values for the remaining demographic and socio-economic variables.

Table 3 shows the estimated coefficients for each regression model in the series of concatenated regressions included in M2.

**Table 3:** Estimated coefficients of the concatenated multivariable regressions from module 2 (M2)

Variables	(1) Gestational age at birth (weeks)	(2) Birth weight (kg)	(3) Weight at 6 months (kg)	(4) Weight at 18 months (kg)	(5) BMI at 48 months	(6) BMI at 84 months	(7) BMI at 120 months
EHII	0.31 [0.11 to 0.51]	− 0.55 [− 0.99 to − 0.10]	− 0.60 [− 1.58 to 0.39]	1.36 [0.07 to 2.65]	− 0.31 [− 0.57 to − 0.05]	− 0.52 [− 0.93 to − 0.10]	− 0.85 [− 1.58 to − 0.12]
Mother’s age	− 0.05 [− 0.06 to − 0.04]	0.03 [0.01 to 0.06]	− 0.05 [− 0.11 to − 0.00]	− 0.02 [− 0.09 to 0.05]	− 0.00 [− 0.02 to 0.01]	0.00 [− 0.02 to 0.03]	− 0.01 [− 0.05 to 0.03]
Mother’s country of birth	0.02 [− 0.19 to 0.22]	0.65 [0.11 to 1.19]	1.88 [0.66 to 3.09]	− 1.30 [− 2.82 to 0.23]	− 0.20 [− 0.54 to 0.14]	− 0.01 [− 0.62 to 0.61]	0.17 [− 0.87 to 1.22]
Sex	− 0.05 [− 0.13 to 0.04]	− 1.39 [− 1.58 to − 1.19]	− 4.46 [− 4.90 to − 4.02]	− 1.47 [− 2.07 to − 0.86]	0.29 [0.18 to 0.41]	0.16* [− 0.01 to 0.34]	− 0.12 [− 0.40 to 0.17]
Gestational age at birth (weeks)		1.70 [1.63 to 1.76]	− 0.46 [− 0.62 to − 0.29]				
Birth weight (kg)			0.89 [0.83 to 0.94]	0.23 [0.16 to 0.30]			
Weight at 6 months (kg)				0.83 [0.79 to 0.87]	0.01 [0.00 to 0.02]		
Weight at 18 months (kg)					0.05 [0.05 to 0.06]	0.02 [0.01 to 0.03]	
Body mass index at 48 months						0.55 [0.48 to 0.63]	0.27 [0.15 to 0.39]
Body mass index at 84 months							0.77 [0.67 to 0.88]
Constant	38.83 [37.39 to 40.27]	− 31.01 [− 34.98 to − 27.03]	73.19 [64.52 to 81.86]	32.69 [22.92 to 42.47]	10.70 [8.75 to 12.66]	8.73 [5.55 to 11.92]	7.62 [2.03 to 13.22]
Observations	6387	6202	5173	4141	2923	1621	658
*R*^2^	0.01	0.37	0.28	0.46	0.20	0.26	0.49

*Note*: 95% confidence intervals in brackets. *EHII* Equivalised Household Income Indicator. Sex (0 = male; 1 = female). Mother’s country of birth (0 = Italy; 1 = others)

The significant reduction in terms of the number of observations over ages was not due to cohort attrition but to the dynamic recruitment of the children from the year 2004 onwards; as a result, most of the children recruited in more recent years have not attained the oldest ages, as explained in Additional file 3.

Table 4 shows the prevalence ratios of overweight and obesity between the baseline scenario and the eight combinations of fiscal interventions according to Table 1, for children of 48, 84 and 120 months of age. Reductions in terms of population prevalence for the results of Table 4 are reported in Additional file 1: Table S3.

**Table 4:** Prevalence ratios and prediction intervals for children overweight and obesity at 48, 84 and 120 months

	Basic income		Poverty reduction		New-borns benefit		Child benefit	
	BI1	BI2	PR1	PR2	NB1	NB2	CB1	CB2
*48 months*								
*Children overweight*	0.994 [0.987–1.000]	0.946 [0.895–0.998]	0.996 [0.991–1.000]	0.967 [0.934–1.001]	0.999 [0.998–1.000]	0.993 [0.985–1.000]	0.993 [0.985–1.001]	0.952 [0.904–1.000]
*Children obesity*	0.986 [0.966–1.005]	0.907 [0.809–1.004]	0.988 [0.971–1.006]	0.936 [0.861–1.012]	0.998 [0.994–1.002]	0.987 [0.971–1.004]	0.982 [0.957–1.008]	0.912 [0.813–1.011]
*84 months*								
*Children overweight*	0.991 [0.985–0.996]	0.913 [0.858–0.968]	0.994 [0.99–0.998]	0.946 [0.911–0.982]	0.999 [0.997–1.000]	0.988 [0.979–0.996]	0.989 [0.982–0.996]	0.921 [0.869–0.974]
*Children obesity*	0.968 [0.94–0.995]	0.807 [0.67–0.945]	0.972 [0.947–0.997]	0.854 [0.734–0.974]	0.996 [0.991–1.002]	0.973 [0.949–0.997]	0.957 [0.918–0.995]	0.805 [0.654–0.957]
*120 months*								
*Children overweight*	0.988 [0.982–0.993]	0.876 [0.819–0.933]	0.992 [0.988–0.996]	0.925 [0.888–0.961]	0.998 [0.997–0.999]	0.982 [0.972–0.992]	0.986 [0.98–0.993]	0.887 [0.831–0.943]
*Children obesity*	0.946 [0.918–0.975]	0.666 [0.501–0.83]	0.951 [0.924–0.979]	0.721 [0.562–0.881]	0.993 [0.985–1.001]	0.950 [0.917–0.982]	0.920 [0.875–0.965]	0.639 [0.439–0.838]

*Note*: 95% confidence intervals in brackets. Ratios and prediction intervals according to the different fiscal reform scenarios in comparison with the baseline

Firstly, we observe that child benefit intervention CB2, consisting of a monthly benefit amount of €500 for each child younger than 5 years in families with household equivalised disposable income lower than €500 has very strong effects on overweight (− 11%) and obesity (− 36%) for children of 120 months of age, with a prevalence rate of 0.89 (95%CI 0.83–0.94) and 0.64 (95%CI 0.44–0.84), respectively. Secondly, basic income intervention BI2, consisting of a monthly benefit amount of €100 for each family member and no further eligibility requirements, again for children of 120 months of age, shows quite important effects on overweight (− 12%) and obesity (− 33%) with a prevalence rate of 0.88 (95%CI 0.82–0.93) for the former outcome variable of interest and 0.67 (95%CI 0.50–0.83) for the latter.

**Fig. 2:**
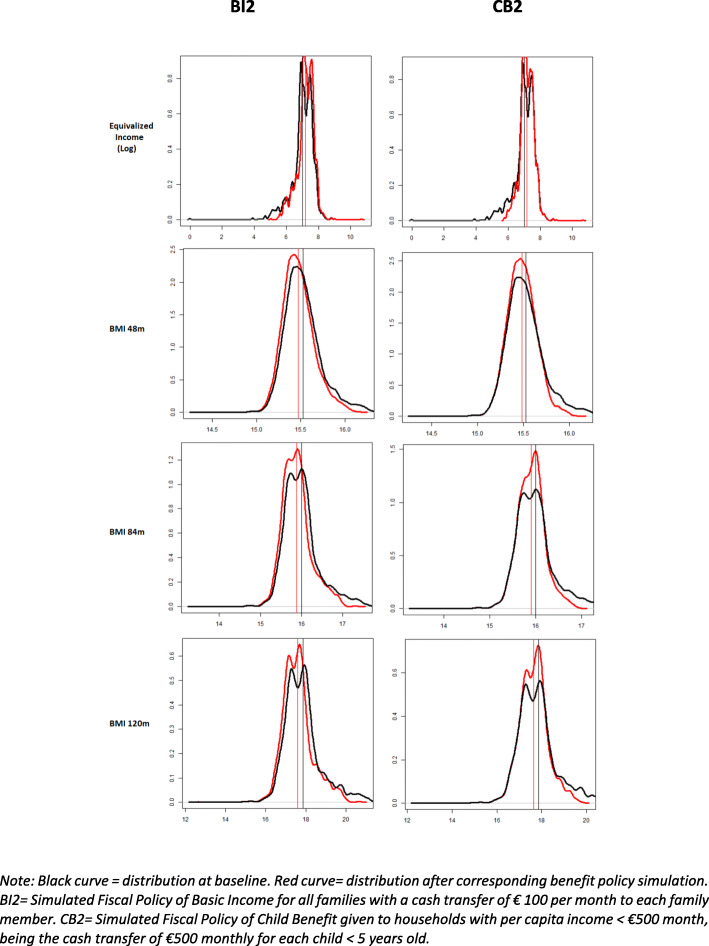
Kernel density plots of the distribution of the logarithm of equivalised disposable income, and BMI at 48, 84 and 120 months of age

Figure 2 shows the distribution of the equivalised disposable income in logarithmic units and the distribution of body mass index (BMI) at different ages. The first column of figures portraits the less focalized intervention, basic income BI2, whereas the second column depicts the more focalized intervention, child benefit CB2. The black curve represents the distribution at the baseline scenario, whereas the red curve represents the distribution after the benefit policy simulation.

Basic income BI2 simulation shows a similar pattern to child benefit CB2 simulation, although shifts for the latter seem to be more distinct than those for the former. Child benefit CB2 simulation shows a correspondence between the shift to the right on the equivalised income distribution and the subsequent shift to the left on BMI distributions at all ages.

Finally, Table 5 shows the estimated costs for a 1% prevalence reduction in overweight and obesity at 48, 84 and 120 months of age with respect to the baseline scenario. It is noteworthy to mention that another salient feature of EUROMOD is that it automatically computes and provides the prospective cost of each simulated scenario. Given the different characteristics of the simulated benefit policies, our estimated cost computations for 1% prevalence reduction show a wide range of values. At 48 months after birth, NB1 and CB1 policies are the most efficient scenarios for both overweight and obesity, with estimated costs of €6.4 and €27.6 billion, respectively, for NB1 and €4.7 and €15.0 billion, respectively, for CB1. At 84 months after birth, the most favourable policies are the same as those at 48 months after birth. NB1 shows costs of €2.4 and €10.7 billion for overweight and obesity, respectively, whereas CB1 depicts costs of €2.0 and €5.5 billion for the aforementioned variables, respectively. Finally, at 120 months after birth, again, NB1 and CB1 scenarios are the most efficient in order to reduce prevalence in 1% with respect to the baseline scenario. For overweigh and obesity, NB1 shows costs of €1.4 and €4.6 billion, respectively, whereas CB1 scenario shows costs of €1.2 and €2.7 billion, respectively.

**Table 5:** Estimated costs for 1% prevalence reduction in overweight and obesity

	Basic income		Poverty reduction		New-borns benefit		Child benefit	
	BI1	BI2	PR1	PR2	NB1	NB2	CB1	CB2
48 months								
Overweight	79.8	109.9	20.6	32.5	6.4	8.7	4.7	8.4
Obesity	272.1	505.0	60.7	133.2	27.6	40.5	15.0	36.4
84 months								
Overweight	33.7	43.1	9.0	12.7	2.4	3.3	2.0	3.3
Obesity	109.0	217.1	22.7	51.5	10.7	17.0	5.5	14.6
120 months								
Overweight	19.7	23.3	5.6	7.0	1.4	1.8	1.2	1.8
Obesity	59.3	110.2	11.9	23.8	4.6	7.9	2.7	6.9

*Note*: Prevalence reduction with respect to the baseline scenario. Figures in billions of euros

It is worthy to point out that, according to the results shown in Tables 4 and 5, we observe that at 48, 84 and 120 months after birth, cost estimates for NB1 and NB2 are distinctly smaller than those corresponding to basic income scenarios BI1 and BI2. However, estimated reductions in prevalence ratios among BI1 and new-borns benefit scenarios, NB1 and NB2, are rather similar.

## Discussion

This study shows how simulated fiscal reforms, and in particular, poverty-reduction fiscal policies, could strongly reduce overweight and childhood obesity in a high-income European country, such as Italy. Our findings quantify also the dose-response relationship between increased benefits and impact on overweight and obesity for each of the eight simulated fiscal interventions. Moreover, focalising the simulated interventions on households with new-borns or children, instead of on all households, seemed to be particularly efficient in terms of marginal benefits.

To our knowledge, this is the first study that creates a comprehensive microsimulation model to evaluate the effects of fiscal policies on health, taking advantage of a consolidated platform such as EUROMOD and integrating it with microsimulation algorithms that project the effects of the equivalised disposable household income on the chosen health outcome. Ultimately, our approach allows to forecast the effectiveness and efficiency of large fiscal interventions on a representative sample of the population.

Besides its methodological sophistication and comprehensiveness, the MICH model constitutes an unprecedented attempt to provide evidence of how fiscal policies could affect health outcomes in the population and could be a systematically used tool in fiscal and health policy-making. Only another recently published study used EUROMOD to evaluate the impact of fiscal policies on overall mortality in Scotland, showing that policies targeting the poorest populations were the most effective to reduce inequalities [30]. However, while its fiscal simulations were comprehensive, the association between income and mortality—used to develop all forecasting scenarios—was not obtained through individual-level longitudinal data, but it was estimated by a cross-sectional regression model based only on population quintiles.

Fiscal reforms have historically been evaluated only in terms of their economic effects, while their impact on other dimensions of society has been neglected [31]. The MICH model could provide evidence of such effects at the time of decision-making, allowing a more balanced and informed choice and implementation of fiscal policies. Moreover, the model is particularly relevant in high-income countries where poverty-alleviation policies are mainly based on fiscal reforms and are often required to demonstrate effective targeting and efficiency of fiscal benefits to the poorest populations. Finally, being based on data from the EUROMOD platform and from the EU-SILC surveys, which are available for several European countries, and being the EHII available for several European birth cohort studies (almost all LifeCycle cohorts [27]), the MICH model could be used to evaluate and compare the effect of a broad range of fiscal policies on childhood health across different European countries.

The choice of the fiscal policies in the simulation has been based on the most common universal and targeted poverty-reduction interventions currently implemented by the majority of EU governments. Household disposable income is among the strongest social determinant of health, because it is a direct measure of material resources, and changes in its levels could have an effect on several health outcomes for the members of the family [2, 32–34]. Previous studies have shown an association between household income and child obesity [35, 36], suggesting the implementation of income subsidies to reduce the economic restrictions of those individuals with lesser economic means. To the best of our knowledge, there are no randomised trials of fiscal interventions or increased total family disposable income with child BMI as the outcome, but a few quasi-experimental studies have evaluated the impact of cash transfers on child obesity [37]. The overall impact of these interventions seems to be dependent on the baseline income of the intervention recipients, the age of children at the time of transfer and also the context where the intervention is delivered [38–40].

Other mathematical modelling studies have attempted to evaluate the effects of different interventions on childhood obesity. A recent microsimulation study showed the combined effectiveness of after-school physical activities, sugar-sweetened beverage taxes and a ban on child-directed fast-food advertising [8]; another study created a specific microsimulation model called Early Prevention of Obesity in Childhood (EPOCH) which was able to model BMI trajectories from early childhood to adolescence [10]. Several other simulation models included behavioural and environmental contributors, and focused policy interventions [9]. However, none of these foregoing studies included income, and its variations due to poverty-reduction interventions, as one of the explanatory variables of childhood obesity.

This study and its microsimulation model are particularly relevant during the current COVID-19 pandemic, which has triggered the worst global economic crisis since the Great Depression, with a World GDP contraction of 4.5% and dramatic increases in unemployment and poverty rates in almost all nations [41, 42].

One of the main instruments used by most governments to increase the resilience of their populations has been extended fiscal benefits but is still unclear how much these interventions have been able to mitigate the increase in household poverty levels. In fact, the strict or partial lockdowns applied in almost all countries could have increased sedentary behaviours, and the dramatic income losses could have forced some families to buy cheaper and more obesogenic foods, especially for children. While physical activity interventions would be difficult and slow to implement due to lockdown measures, fiscal poverty-reduction interventions could be a fast and effective instrument to curb this tendency.

One of the main limitations of this study regards the estimated marginal effects. Fiscal reforms have a wide range of effects, spanning from socio-economic to health outcomes, and the objective of our study is to show their—mostly unintended—impact only on a specific health outcome, child overweight and obesity. As a consequence, the exercise of evaluating the marginal benefits cannot be interpreted as a cost-effectiveness evaluation. Another limitation of our study is that, while our statistical associations, obtained from a cohort study, are respecting many of the traditional conditions of causality (strength of the associations, consistency, specificity, temporality and plausibility), and have the advantage of being specific for the population in which the simulations were conducted, the observational nature of the study cannot rule out the possibility that they are not causal. We also acknowledge that the NINFEA cohort has attrition, even if within the standard magnitude for such kind of child cohorts. Moreover, we did not include all the variables associated with overweight and obesity in the concatenated regression models of the MICH M2 module. This is because we included confounding factors only of the association between EHII and BMI and not mediators of such association (such as childhood diet, physical activity) that would have affected the real effect estimates of EHII. However, due to the complexity of the overweight and obesity theoretical framework, we acknowledge that we cannot completely rule out the possibility of remaining omitted variable bias.

## Conclusions

In conclusion, our study illustrates the construction of an unprecedented integrated microsimulation model—based on the consolidated EUROMOD platform—able to forecast the effect of a broad range of fiscal interventions on childhood obesity, with algorithms and codes potentially flexible to be used for other health outcomes and for other European countries. While the impact of fiscal policies is usually measured on economic outcomes, our study was the first to quantify their effects on one of the most concerning child health problems in high-income countries. Potential impacts of fiscal interventions on the health of the population should be taken into account during the process of policy-making and should be considered in the framework of the Health in All (HiAP) policies [11].

## Supplementary Information

**Additional file 1.** Additional description of the modelling process.

**Additional file 2.** Additional information about the European tax-benefit model EUROMOD.

**Additional file 3.** Additional information about the NINFEA cohort.

## Data Availability

Anonymus individual data from the NINFEA Cohort could be provided upon request to the authors. The EU-SILC dataset used as input for EUROMOD is available upon request to Eurostat (https://ec.europa.eu/eurostat) and to EUROMOD (https://euromod-web.jrc.ec.europa.eu/)

## Abbreviations

BI: Universal basic income
BI1: Baseline income scenario 1
BI2: Baseline income scenario 2
BMI: Body mass index
BMI_120m_: Child’s body mass index at 10 years of age
BMI_48m_: Child’s body mass index at 4 years of age
BMI_84m_: Child’s body mass index at 7 years of age
BS: Baseline scenario
BW: Child’s birth weight
CB: Child benefit
CB1: Child benefit scenario 1
CB2: Child benefit scenario 2
CEDAP: Certificato di Assistenza al Parto (translated as Italian Birth Registry)
COVID-19: Coronavirus disease 2019
DG EMPL: Directorate-General for Employment, Social Affairs and Inclusion
EHII: Equivalised Household Income Indicator
EPOCH: Early Prevention of Obesity in Childhood
EU: European Union
EU-SILC: European Union Statistics on Income and Living Conditions
GA: Gestational age
GDP: Gross domestic product
H2020: Horizon 2020
HiAP: Health in All Policies
IOTF: International Obesity Task Force
ISPOR-SMDM: International Society for Pharmacoeconomics and Outcomes Research and the Society for Medical Decision Making
M1: Module 1
M2: Module 2
M3: Module 3
MICH: Microsimulation for Income and Child Health
NB1: New-borns benefit scenario 1
NB2: New-borns benefit scenario 2
NINFEA: Nascita e Infanzia: gli Effetti dell’Ambiente (translated as Birth and Childhood: the Effects of the Environment)
OECD: Organisation for Economic Cooperation and Development
PR: Prevalence ratio
PR1: Poverty reduction scenario 1
PR2: Poverty reduction scenario 2
SEP: Socio-economic position
UK: United Kingdom
WT_18m_: Child’s weight at 18 months after being born
WT_6m_: Child’s weight at 6 months after being born
