# Supplementary material for: Developing an integrated microsimulation model for the impact of fiscal policies on child health in Europe: the example of childhood obesity in Italy
Source: BMC Med. 2021 Nov 30;19:310. doi: 10.1186/s12916-021-02155-6 (PMC8629597; doi:10.1186/s12916-021-02155-6)
Supplement: Supplementary file 1 — Additional file 1. Additional description of the modelling process. [file 12916_2021_2155_MOESM1_ESM.docx]

**Additional file 1**

**Supplement to: Developing an integrated microsimulation model for the impact of fiscal policies on child health in Europe: the example of childhood obesity in Italy**

Davide Rasella, PhD ^1,2^; Lorenzo Richiardi, PhD ^2^; Nicolai Brachowicz, MSc ^1^, H. Xavier Jara, PhD ^3^; Mark Hanson, PhD ^4^; Delia Boccia, PhD ^2,5^; Matteo G. Richiardi, PhD ^3^; Costanza Pizzi, PhD ^2^

^1^ ISGlobal, Hospital Clínic - Universitat de Barcelona, Barcelona, Spain

^2^ Department of Medical Sciences, University of Turin, Turin, Italy

**^3^** Centre for Microsimulation and Policy Analysis, Institute for Social and Economic Research, University of Essex, Colchester, UK

^4^ Institute of Developmental Sciences and NIHR Biomedical Research Centre, University of Southampton and University Hospital Southampton, UK.

^5^ Faculty of Population and Health Policy, London School of Hygiene and Tropical Medicine, London, United Kingdom

**DESCRIPTION OF THE MODELLING PROCESS**

The following section provides details of each of these processes in accordance with standard international modelling reporting guidelines (ISPOR-SMDM).

**Purpose of the model and its applications**

The developed model, named Microsimulation for Income and Child Health (MICH), aims to simulate the effects of fiscal policy reforms using the EUROMOD platform combined with health cohorts - health surveys - with a measure of income associated with the child health outcome of interest.

In this first pilot study we used MICH to forecast the effects of a range of fiscal policies on childhood overweight and obesity in Italy, evaluating the efficiency and effectiveness of universal basic income versus more targeted poverty-reduction interventions.

**Theoretical framework of the model**

Household disposable income is among the strongest social determinant of health, because it is a direct measure of material resources, and changes in its levels could have an effect on several health outcomes for the members of the family. While the effect of income changes can be different in high and middle and low-income countries, previous studies have shown an association between household income and child obesity, suggesting the implementation of income subsidies to improve the economic restrictions of those individuals with lesser economic means.

**Overall Model Structure**

The modelling strategy involves three integrated modules. The first module (M1 – the tax-benefit microsimulation model) uses EUROMOD to simulate the effects on EU-SILC data of different fiscal reforms on the equalized disposable income of all households with at least one child aged less than 5 years. The second module (M2 – the concatenated regressions model) exploits the information on the EU-SILC equivalised total disposable household income as obtained in M1 and the data of the Italian NINFEA birth cohort study, for which Body Mass Index (BMI) at different ages in childhood and other intermediate outcomes - namely Gestational Age (GA), Birth Weight (BW) and Weight during infancy (WT) - are available, to estimate the effects of the household income on these outcomes. The third module (M3 – the integrated microsimulation model) combines the equivalised total disposable incomes obtained from M1 with the regression model structures, specifications and effect sizes from M2, to simulate the distribution of BMI at different ages in childhood according to the specific fiscal reform implemented. The overall model structure, which we call Microsimulation for Income and Child Health (MICH), and flow of inputs and outputs for each stage are shown in Figure 1.

*M1: EUROMOD and the Fiscal Reform Scenarios*

EUROMOD is a tax-benefit microsimulation model for the European Union that enables researchers to calculate, in a comparable manner, the effects of taxes and benefits on household incomes for the population of each country and for the EU as a whole. It is one of the oldest and more consolidated microsimulation models in the EU and its focus is on fiscal policies, having been extensively used in economic and social studies. We use the 2010 wave of EU-SILC-Italy as the input population for the EUROMOD, to be consistent with the income values of the NINFEA cohort of 2011 used for the M2 regression models. No EU-SILC-Italy as EUROMOD input was available for 2011. As the baseline policy system against which we evaluate the effectiveness of alternative policies, we use the policy rules in place in June 2018, as the latest available at the time of this study.

**Figure S1 Structure of the MICH Model: flow of inputs and outputs data and parameters between M1, M2 and M3 modules.**

*Note: For M1: data provided by the Italian EU-SILC 2010 survey. Fiscal Policies simulated using EUROMOD software. For M2: data provided by NINFEA cohort. Concatenated regressions run in Stata. For M3: dynamic microsimulation using R.*

The model produces as output a population that is the same as the EU-SILC sample (46,788 individuals), but with added information on disposable income for each individual, based on the specific policy system (actual or hypothetical) considered. For this project we aggregate individual disposable income at the household level, and then adjust it to account for household size and composition, using the so-called modified OECD equivalence scale. For reasons of comparability with the NINFEA cohort analyses, we excluded families larger than 7 members and families with no children less than 5 years old.

EUROMOD also provides the overall cost for the public budget, and therefore, after comparison with the baseline, the cost of the changes implemented in the counterfactual scenarios, which can be used to calculate the marginal benefit (health outcome gain compared with the policy cost) of the reforms.

*M2: The Concatenated Regression Models in the NINFEA birth cohort*

NINFEA is and Italian web-based birth cohort study, which recruited pregnant women from 2005 to 2016 and follows up their children via online questionnaires completed by the mothers at 6 and 18 months after delivery and when children turn 4, 7, 10 and 13 years of age. For this paper we used the NINFEA database version 02.2019 that consists of 6625 mothers and 7423 pregnancies.

Data on demographic and socioeconomic factors of the family are collected with the baseline questionnaire completed during pregnancy. Child weight and height data, used to derive the BMI, are collected at each follow-up questionnaire. As described in detail in the paper by Pizzi et al., an indicator of the EU-SILC-based equivalised total disposable household income (the Equivalised Household Income Indicator (EHII)) has been derived for the NINFEA participants within the framework of the H2020 LifeCycle project. In brief the EHII was derived using external data from the Italian 2011 EU-SILC survey and individual and household characteristics available in the NINFEA cohort (namely parental age, cohabitation status, education, country of birth and occupation, house size and type and family size). The EHII is the log transformation of such equivalised total disposable household income as used in the original paper.

*M3: The integrated microsimulation model*

The last module of MICH applies the effects sizes from M2 to the population from M1. From the EUROMOD output we select the population of children less than 5 years old, and expand it using the EUSILC-Italy survey sample weights, obtaining a study population of 30,910 children.

Using the same set of concatenated multivariable linear regressions shown above, with the outputs from M1 and M2 used as inputs and the alphas, betas and standard errors of the regressions from M2, the integrated model estimates the distribution of gestational age for the population of children under 1 year old using regression n.1. It is worthy to notice that the betas and alphas have been obtained from the matrix of variance-covariance obtained through the R command vcov, and the combination of intercepts and betas for all 1000 simulations through the R command mvrnorm.

The obtained distribution of gestational ages is successively introduced in regression n.2 together with the same set of demographic and socioeconomic factors – including the equivalised income – from M1. The regressions from 3 to 7 use the same principle, creating a flow of outputs used as inputs for the following models, and allowing us to simulate the final BMI distributions at 18, 48 and 84 months.

**Simulation of Fiscal Intervention Scenarios**

Fiscal reform scenarios were conceived with an increased degree of focalization to evaluate the effectiveness of targeting the most vulnerable, and each policy was implemented with two levels of benefit amounts – with the same eligibility rules and the same recipients – to evaluate the dose-response effect. This design therefore allowed us to evaluate different degrees of marginal benefits according to intervention focalization and intensity. The simulated fiscal interventions are shown in Table 1. While the baseline, used for comparison with all the other scenarios, is produced applying the actual 2018 fiscal regime on the EU-SILC survey data, the Basic Income Scenario applies a Basic Income of 100 Euros per year (BI1) or per month (BI2) to all citizens, and the Poverty Reduction scenarios simulate a poverty-relief intervention of 100 Euros per year (PR1) or per month (PR2) for each member of the households with an equivalised income of less than 500 Euros per month. The New-Born Benefit is a simulated fiscal intervention of 500 Euros per year (NB1) or per month (NB2) for each child less than one year old in households with an equivalised income of less than 500 Euros per month. The Child Benefit (CB) simulated policy has the same characteristics as the New-born benefits but the recipients are each children of less than 5 years (instead of less than 1 year) of age, reaching a larger number of households than the NB benefits. It is important to note that these benefit amounts are not chosen as realistic values, nor they can be directly compared across the different policy scenarios, and leading to very different total costs for the public budget. However, these scenarios allow us to compute marginal benefits, which are a normalised measure of the effectiveness of the different policy instruments, and can be used to compare the health effects of each alternative.

|  | Baseline (BS) | Basic Income (BI) | | Poverty Reduction (PR) | | New-borns Benefit (NB) | | Child Benefit (CB) | |
| --- | --- | --- | --- | --- | --- | --- | --- | --- | --- |
|  |  | BI1 | BI2 | PR1 | PR2 | NB1 | NB2 | CB1 | CB2 |
| Eligibility | - | all | all | households with per capita income  < €500 month | households with per capita income  < €500 month | households with per capita income  < €500 month | households with per capita income  < €500 month | households with per capita income  < €500 month | households with per capita income  < €500 month |
|  |  |  |  |  |  |  |  |  |  |
|  |  |  |  |  |  |  |  |  |  |
|  |  |  |  |  |  |  |  |  |  |
| Benefit Amount | - | € 100 | € 100 | € 100 | € 100 | € 500 | € 500 | € 500 | € 500 |
|  |  |  |  |  |  |  |  |  |  |
|  |  |  |  |  |  |  |  |  |  |
|  | - | yearly | monthly | yearly | monthly | yearly | monthly | yearly | monthly |
| Periodicity |  |  |  |  |  |  |  |  |  |
|  |  |  |  |  |  |  |  |  |  |
| Recipients | - | all household members | all household members | all household members | all household members | every child < 1 year old | every child < 1 year old | every child < 5 years old | every child < 5 years old |
|  |  |  |  |  |  |  |  |  |  |
|  |  |  |  |  |  |  |  |  |  |

**Table S1 Simulated tax-benefit scenarios**

*Note: Each simulated fiscal policy has two levels of intensity keeping other features fixed. The same benefit amount is given once a year or once a month.*

**Figure S2 Kernel Density plots of the distribution of the logarithm of equivalised disposable income, and BMI at 48, 84, and 120 months of age**

**
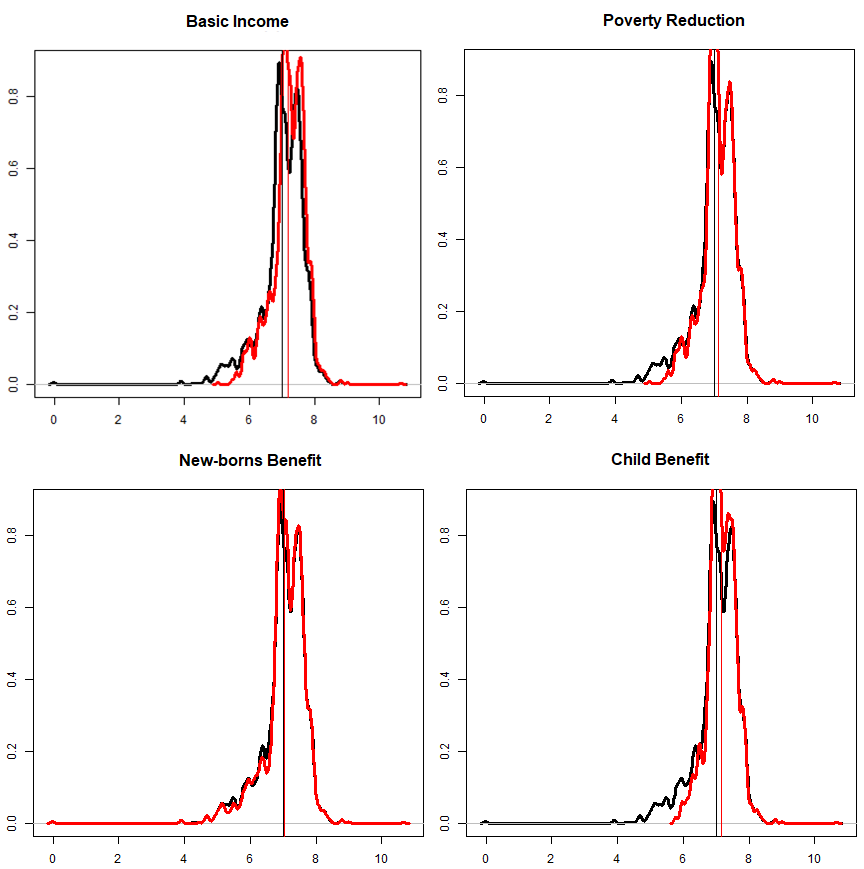
**

*Note: Black curve = distribution at baseline. Red curve= distribution after corresponding benefit policy simulation.*

**Data sources, inputs, outputs, and other parameters**

The structure of the concatenated regressions models used in M2 and fitted on the NINFEA data is described in the following equations:

*1) GA = α + δEHII + Σ β_s_ X_s_ + ε*

*2) BW=* *α + β_1_GA + δEHII + Σ β_s_ X_s_ + ε*

*3) WT_6m_= α + β_1_BW + β_2_GA + δEHII + Σ β_s_ X_s_ + ε*

*4) WT_18m_= α + β_1_WT_6m_ + β_2_BW + δEHII + Σ β_s_ X_s_ + ε*

*5) BMI_48m_= α + β_1_WT_18m_* + *β_2_WT_6m_ + δEHII + Σ β_s_ X_s_ + ε*

*6) BMI_84m_= α + β_1_BMI_48m_ + β_2_WT_18m_ + δEHII + Σ β_s_ X_s_ + ε*

*7) BMI_120m_= α + β_1_BMI_84m_ + β_2_BMI_48m_ + δEHII + Σ β_s_ X_s_ + ε*

with GA (gestational age) as the first intermediate outcome analysed, followed by BW (birth weight), WT (weight) at 6 and 18 months, and then by the final outcomes: BMI at 4, 7 and 10 years of age. In all equations EHII is the income indicator (log-transformed), with δ being the coefficient of interest for the income indicator. Moreover, α is the intercept – different for each regression - *Σ β_s_ X_s_* is the sum of the other demographic considered, namely the sex of child, and the maternal country of birth and age at delivery, and *ε* is the error component. The underlying assumption is that all outcomes analysed are influenced by the two previous ones and by the other factors cited above. These models provide for each independent variable the effect sizes, with their corresponding confidence intervals and covariance matrixes, to be used in the microsimulation model M3.

Figure 3 shows the comparison between the distribution of equivalised income, age and citizenship of the mother, and sex of the children in the EU-SILC dataset of children less than 5 years and the children in NINFEA cohort.

The simulated baseline demographic and socioeconomic variables derived from MICH are shown in Table 2, as well as the health outcomes and all variables and parameters used to obtain them in M1 and M2. The log of the household equivalised income at the baseline is lower in the EU-SILC population than in the NINFEA cohort (the mean is 7.02 vs 7.38), while the percentage of mothers not born in Italy are higher in EU-SILC (16.8%) than in NINFEA (5.1%). The other demographic and socioeconomic variables have similar values.

| **Table S2 Estimated means, percentages and parameters used in the M1, M2 and M3 modules of the MICH** **model** | | | |
| --- | --- | --- | --- |
|  |  |  |  |
|  | M1 | M2 | M3 |
|  | EUROMOD (EU-SILC) | NINFEA Cohort | Baseline Simulated Values* |
| Health Outcomes |  |  |  |
| Gestational Age | - | 39.5[1.8] | 39.6[0.28] |
| Birth Weight | - | 3,237[499] | 3,218[66] |
| Weight at 6 months | - | 7,539[942] | 7,774[241] |
| Weight at 18 months | - | 11,162[1,285] | 11,286[256] |
| BMI at 48 months | - | 15.6[1.7] | 15.5[0.2] |
| BMI at 84 months | - | 15.9[2.1] | 16.0[0.5] |
| BMI at 120 months | - | 17.3[2.6] | 17.9[1.0] |
| Demographic and Socioeconomic Predictors |  |  |  |
|  |  |  |  |
| Female gender | 51.60% | 49.30% | - |
| Log of Equivalised income | 7.02 [0.67] | 7.38 [0.26] | - |
| Foreign citizenship of the mother | 16.80% | 4.20% | - |
| Age of the mother | 33.6 [5.2] | 33.3[4.4] | - |
| *Note: Weights in kg. Estimated mean values (with standard deviations in brackets), or percentages. *Distribution of the means of the total runs.* | | | |
|  | | | |

Scenarios were compared in terms of prevalence ratios (using the real fiscal scenario as denominator) and marginal benefits were obtained by dividing the cost of the fiscal intervention - provided by the EUROMOD module - by the prevalence difference between scenarios.

**Figure S3 Kernel Density of the distribution of demographic and socioeconomic covariates between the EU-SILC population of children with less than 5 years (Blue) and the NIFEA children cohort (red).**


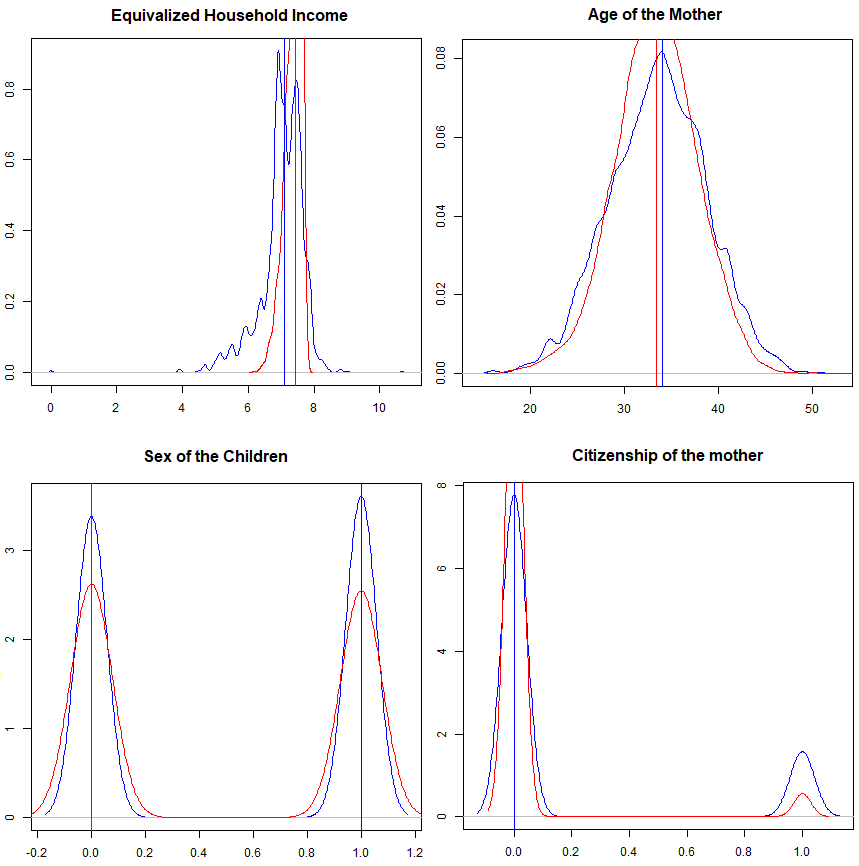


*Source: Author’s.*

**Summary of Results (more broadly explained in the manuscript)**

Table 3 shows prevalence of children’s overweight and obesity for baseline and each simulated policy scenario at 48, 84, and 120 months after birth. Table 4 shows prevalence ratios of obesity (PR) between the baseline scenario and the eight combinations of fiscal interventions coded according to Table 1. These results indicate that the strongest effects on obesity are found at 120 months in case of the targeted intervention of Child benefits of 500 Euro per each month for each child younger than 5 years in families with household equivalised income lower than 500 Euro - CB2, with a PR of 0.64 (95%CI: 0.44-0.84); and with the Basic Income of 100 Euro per month for each member of the family - BI2 (PR 0.67; 95%CI: 0.50-0.83). The strongest effects on overweight and obesity are also found in CB2 (PR 0.89; 95%CI: 0.83-0.94) and BI2 (PR 0.88; 95%CI: 0.82-0.93).

**Table S3 Prevalence of children’s overweight and obesity for baseline and each simulated policy scenario at 48, 84, and 120 months after birth**

|  | Baseline | Base Income | | Poverty Reduction | | New-borns Benefit | | Child Benefit | |
| --- | --- | --- | --- | --- | --- | --- | --- | --- | --- |
|  |  | BI1 | BI2 | PR1 | PR2 | NB1 | NB1 | CB1 | CB2 |
| At 48 months |  |  |  |  |  |  |  |  |  |
| overweight | 11.980 | 11.905 | 11.325 | 11.928 | 11.580 | 11.970 | 11.890 | 11.893 | 11.394 |
| obesity | 1.463 | 1.441 | 1.321 | 1.445 | 1.365 | 1.461 | 1.444 | 1.436 | 1.328 |
| At 84 months |  |  |  |  |  |  |  |  |  |
| overweight | 18.897 | 18.719 | 17.227 | 18.777 | 17.873 | 18.870 | 18.659 | 18.690 | 17.390 |
| obesity | 1.643 | 1.588 | 1.311 | 1.595 | 1.390 | 1.637 | 1.597 | 1.568 | 1.306 |
| At 120 months |  |  |  |  |  |  |  |  |  |
| overweight | 24.728 | 24.424 | 21.642 | 24.535 | 22.859 | 24.680 | 24.278 | 24.389 | 21.922 |
| obesity | 1.879 | 1.778 | 1.225 | 1.788 | 1.331 | 1.865 | 1.779 | 1.727 | 1.168 |

*Source: Author’s.*

**Table S4 Estimated coefficients of the concatenated multivariable regressions from Module 2 (M2)**

|  | (1) | (2) | (3) | (4) | (5) | (6) | (7) |
| --- | --- | --- | --- | --- | --- | --- | --- |
|  | Gestational age | Birth | Weight | Weight | BMI | BMI | BMI |
| VARIABLES | at birth | weight (kg) | at 6 months (kg) | at 18 months (kg) | at 48 months | at 84 months | at 120 months |
|  |  |  |  |  |  |  |  |
| EHII | 0.31 | -0.55 | -0.60 | 1.36 | -0.31 | -0.52 | -0.85 |
|  | [0.11 - 0.51] | [-0.99 - -0.10] | [-1.58 - 0.39] | [0.07 - 2.65] | [-0.57 - -0.05] | [-0.93 - -0.10] | [-1.58 - -0.12] |
| Mother's age | -0.05 | 0.03 | -0.05 | -0.02 | -0.00 | 0.00 | -0.01 |
|  | [-0.06 - -0.04] | [0.01 - 0.06] | [-0.11 - -0.00] | [-0.09 - 0.05] | [-0.02 - 0.01] | [-0.02 - 0.03] | [-0.05 - 0.03] |
| Mother's country of birth | 0.02 | 0.65 | 1.88 | -1.30 | -0.20 | -0.01 | 0.17 |
|  | [-0.19 - 0.22] | [0.11 - 1.19] | [0.66 - 3.09] | [-2.82 - 0.23] | [-0.54 - 0.14] | [-0.62 - 0.61] | [-0.87 - 1.22] |
| Sex | -0.05 | -1.39 | -4.46 | -1.47 | 0.29 | 0.16* | -0.12 |
|  | [-0.13 - 0.04] | [-1.58 - -1.19] | [-4.90 - -4.02] | [-2.07 - -0.86] | [0.18 - 0.41] | [-0.01 - 0.34] | [-0.40 - 0.17] |
| Gestational age at birth (weeks) |  | 1.70 | -0.46 |  |  |  |  |
|  |  | [1.63 - 1.76] | [-0.62 - -0.29] |  |  |  |  |
| Birth weight (kg) |  |  | 0.89 | 0.23 |  |  |  |
|  |  |  | [0.83 - 0.94] | [0.16 - 0.30] |  |  |  |
| Weight at 6 months (kg) |  |  |  | 0.83 | 0.01 |  |  |
|  |  |  |  | [0.79 - 0.87] | [0.00 - 0.02] |  |  |
| Weight at 18 months (kg) |  |  |  |  | 0.05 | 0.02 |  |
|  |  |  |  |  | [0.05 - 0.06] | [0.01 - 0.03] |  |
| Body Mass Index at 48 months |  |  |  |  |  | 0.55 | 0.27 |
|  |  |  |  |  |  | [0.48 - 0.63] | [0.15 - 0.39] |
| Body Mass Index at 84 months |  |  |  |  |  |  | 0.77 |
|  |  |  |  |  |  |  | [0.67 - 0.88] |
| Constant | 38.83 | -31.01 | 73.19 | 32.69 | 10.70 | 8.73 | 7.62 |
|  | [37.39 - 40.27] | [-34.98 - -27.03] | [64.52 - 81.86] | [22.92 - 42.47] | [8.75 - 12.66] | [5.55 - 11.92] | [2.03 - 13.22] |
|  |  |  |  |  |  |  |  |
| Observations | 6,387 | 6,202 | 5,173 | 4,141 | 2,923 | 1,621 | 658 |
| R-squared | 0.01 | 0.37 | 0.28 | 0.46 | 0.20 | 0.26 | 0.49 |

*Note: 95% Confidence Intervals in brackets. EHII stands for Equivalised Household Income Indicator. Sex (0=Male; 1 Female). Mother’s country of birth (0=Italy; 1= others)*

Distribution of each dependent variable produced by M3 microsimulation models changed between baseline and fiscal intervention scenarios (Figure 4).

**Calibration**

Because the intercepts (Alphas) are obtained from the regression models applied to the NINFEA cohort, which is not representative of the Italian population, for the microsimulation model M2 we needed to calibrate their values. The calibration was achieved by varying the Alpha of the regression n.1 described above, in order to obtain the lower sum of squared errors (SSE) in comparison with the Italian national prevalence of premature births, and for regression n.2 with the average birth weight, both from the Italian birth registry “Certificato di Assistenza al Parto” (CEDAP) of the year 2011.

**Uncertainty Measures**

For each outcome and each scenario, 1,000 simulations were performed using the Monte Carlo sampling method. This allows the main parameter values, in our case the Alphas and Betas of the regression equations, to vary in each simulation cycle according to their assumed underlying distributions and their matrix of variance-covariance. The number of simulations was chosen after evaluating when estimates were stabilizing and further runs were not improving the point estimates, their standard errors and any other aspect of the simulation.

**Figure S4 Kernel Density of the distribution of child health outcomes from the M3 regression model for the Baseline (black) and Basic Income of 100 Euro per month (red).**

**
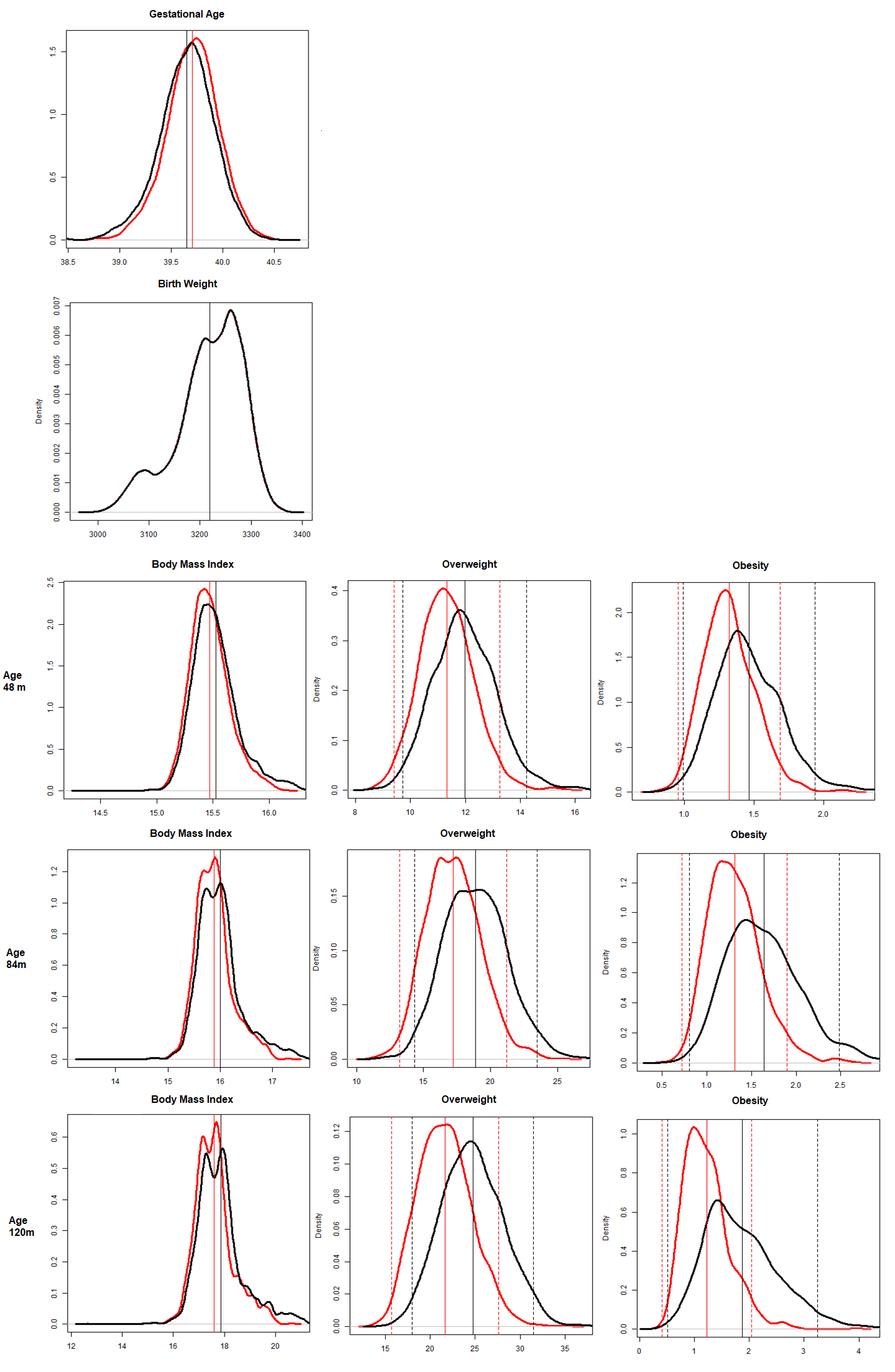
**

*Source: Author’s.*

**Limitations**

One of the main limitations of the study is related to the estimated marginal effects: fiscal reforms have a wide range of effects, spanning from socioeconomic to health outcomes, and the objective of our study is to show their - mostly unintended - impact only on a specific health outcome, child overweight and obesity. As a consequence, the exercise of evaluating the marginal benefits cannot be interpreted as a cost-effectiveness evaluation.

Another limitation is the absence of behavioural and physical activity variables, that could be related with changes in income or socioeconomic position, for the modelling of childhood BMIs. While this has not be introduced in the microsimulation models, we assume that the relationship between income and weight and height measured in the NINFEA cohort is also able to include such more proximal determinants of overweight and obesity.

**Coding Software**

The M1 module was executed in EUROMOD version 3.0.0, its outputs processed in STATA version 14, and M2 and M3 were coded and implemented in R version 3.6.3.
