# Supplementary material for: Developing an integrated microsimulation model for the impact of fiscal policies on child health in Europe: the example of childhood obesity in Italy
Source: BMC Med. 2021 Nov 30;19:310. doi: 10.1186/s12916-021-02155-6 (PMC8629597; doi:10.1186/s12916-021-02155-6)
Supplement: Supplementary file 2 — Additional file 2. Additional information about the European tax-benefit model EUROMOD. [file 12916_2021_2155_MOESM2_ESM.docx]

**Additional file 2**

**Supplement to: Developing an integrated microsimulation model for the impact of fiscal policies on child health in Europe: the example of childhood obesity in Italy**

Davide Rasella, PhD ^1,2^; Lorenzo Richiardi, PhD ^2^; Nicolai Brachowicz, MSc ^1^, H. Xavier Jara, PhD ^3^; Mark Hanson, PhD ^4^; Delia Boccia, PhD ^2,5^; Matteo G. Richiardi, PhD ^3^; Costanza Pizzi, PhD ^2^

^1^ ISGlobal, Hospital Clínic - Universitat de Barcelona, Barcelona, Spain

^2^ Department of Medical Sciences, University of Turin, Turin, Italy

**^3^** Centre for Microsimulation and Policy Analysis, Institute for Social and Economic Research, University of Essex, Colchester, UK

^4^ Institute of Developmental Sciences and NIHR Biomedical Research Centre, University of Southampton and University Hospital Southampton, UK.

^5^ Faculty of Population and Health Policy, London School of Hygiene and Tropical Medicine, London, United Kingdom

**THE EUROPEAN TAX-BENEFIT MODEL EUROMOD**

EUROMOD is a multi-country tax-benefit microsimulation model for the 27 member-states of the European Union and the UK, based on representative household micro-data. In essence, tax-benefit microsimulation models such as EUROMOD are programmes which compute taxes paid and social insurance contribution and benefits received by households, depending on their market incomes and demographic characteristics. Based on microdata that is representative of the population, these models can assess the redistributive and budgetary effects of actual and counterfactual policies.

EUROMOD is a static microsimulation model, meaning that the arithmetic simulations of tax-benefit policies do not consider behavioural responses of individuals or changes in the sociodemographic characteristics of the population over time. However, the results of the EUROMOD simulations can be used as inputs for more advanced models, for example, behavioural models of labour supply or macroeconomic models.

The EUROMOD modelling platform has a flexible and transparent design which allows different country specific tax-benefit systems to be modelled in a common conceptual and technical framework (EUROMOD 2018), with the aim to maximize cross-country comparability. EUROMOD baseline simulations refer to policies as of 30th June of each year in all countries. Aggregate estimates for expenditure (revenue) and total number of benefit recipients (taxpayers) of each benefit (tax) simulated in EUROMOD are validated against official statistics on a yearly basis in the EUROMOD Country Reports. The simulated income distribution and corresponding poverty and inequality indicators are also compared against those of the surveys used as inputs in the simulations. This comparison shows an appropriate EUROMOD performance.

EUROMOD is unique in that it is openly accessible for non-commercial use, with EU-SILC data access subject to permission by Eurostat. EUROMOD has been widely applied in academic research and policy analysis. Due to its flexibility, the EUROMOD platform has also been successfully used to build tax-benefit microsimulation models for non-EU countries.

The EUROMOD platform consists of four main elements: (i) the EUROMOD software, (ii) the tax-benefit policy code for the 27 member-states of the European Union and the UK, (iii) the harmonised input data for the simulations, (iv) detailed modelling guidelines and documentation related to the model.

The *EUROMOD software* consists of a specific programming language for the simulation of taxes and benefits. The EUROMOD software includes the user interface, the executable and a built-in help menu. All these components are generic and independent of the policy simulation codes of each country.

The *tax-benefit policy code* is stored in xml format but can be accessed through the user interface. The policy code in EUROMOD is made up of a series of commands known as functions which are the building blocks for implementing a public policy instrument, for example a tax or a benefit. Each function in EUROMOD contains a series of parameters that represent a specific element of the policy's functionality (e.g. a tax threshold or a tax rate, or a benefit amount). The tax-benefit policy code is specific to each country (i.e. each country has a separate xml file) but implemented in a common programming language.

The *harmonised EUROMOD input data* are derived, for most countries, from the EU-SILC data as released by Eurostat (Eurostat, 2020). In some countries, such as Italy, the national version of SILC is used directly to generate the EUROMOD input data due to the availability of more detailed variables for the simulations. In the specific case of the UK, the Family Resources Survey is used as the microdata source to derive the EUROMOD input data. Based on the original microdata, the EUROMOD methodology consists of creating harmonised datasets that are used as input in the simulation platform. The EUROMOD conventions specify standardised definitions and labelling for variables in the input as well as output databases of the models (EUROMOD 2018). This standardization allows the user to recognize similar variables of income, taxes and benefits in any model that has been developed in the EUROMOD platform and facilitates the use of models for comparative analysis. The EUROMOD input data are accompanied of a Data Requirements Document (DRD), which provides information of the variables included in the data and a description of the derivation from the original data.

*Detailed documentation* of the different stages of the EUROMOD simulations is made available for users. The EUROMOD Modelling Conventions (EUROMOD 2018) collects the rules and guidelines followed by developers in updating the tax-benefit microsimulation model, EUROMOD. Among others, it provides information about the protocols to produce and document the input data, the scope of the policy simulations, and standard assumptions for the simulation of specific policies across countries. The EUROMOD Country Reports contain a detailed description of the tax-benefit instruments simulated in EUROMOD and the specific assumptions in the simulations of these components in each country. They also provide information about any adjustments or imputations made to produce the input data, and the validation of aggregate EUROMOD results compared to external statistics. Annual reports documenting the baseline EUROMOD distributional indicators and discussing discrepancies with those obtained by Eurostat are also produced after every public release of the model.

The EUROMOD platform aims to provide the greatest flexibility for the analysis of the simulation results. For this reason, the final product of the simulation in the model is an output microdata which contains all the variables of the input microdata and additionally includes all the variables simulated in the model. In this way, the user can make use of the output microdata in any statistical program of her choice (for example, Stata, SPSS, R, etc.) to analyse them.
