# Supplementary material for: Developing an integrated microsimulation model for the impact of fiscal policies on child health in Europe: the example of childhood obesity in Italy
Source: BMC Med. 2021 Nov 30;19:310. doi: 10.1186/s12916-021-02155-6 (PMC8629597; doi:10.1186/s12916-021-02155-6)

**Additional file 3**

**Supplement to: Developing an integrated microsimulation model for the impact of fiscal policies on child health in Europe: the example of childhood obesity in Italy**

Davide Rasella, PhD ^1,2^; Lorenzo Richiardi, PhD ^2^; Nicolai Brachowicz, MSc ^1^, H. Xavier Jara, PhD ^3^; Mark Hanson, PhD ^4^; Delia Boccia, PhD ^2,5^; Matteo G. Richiardi, PhD ^3^; Costanza Pizzi, PhD ^2^

^1^ ISGlobal, Hospital Clínic - Universitat de Barcelona, Barcelona, Spain

^2^ Department of Medical Sciences, University of Turin, Turin, Italy

**^3^** Centre for Microsimulation and Policy Analysis, Institute for Social and Economic Research, University of Essex, Colchester, UK

^4^ Institute of Developmental Sciences and NIHR Biomedical Research Centre, University of Southampton and University Hospital Southampton, UK.

^5^ Faculty of Population and Health Policy, London School of Hygiene and Tropical Medicine, London, United Kingdom

**THE NINFEA COHORT**

The NINFEA (Nascita e Infanzia: gli Effetti dell’Ambiente) study is an Italian web-based birth cohort study recruiting pregnant women, started in 2005 in the city of Turin and then extended to the rest of Italy. The study aims at investigating the effect of several exposures acting during pre-natal and early post-natal life on later health ([www.progettoninfea.it](http://www.progettoninfea.it)). Recruitment has been carried out during the period 2005–2016, with the study advertised both actively, through obstetrics clinics, and passively, via Internet and the media. Members of the cohorts are children born to mothers who have access to the internet, have enough knowledge of Italian language to complete online questionnaires and volunteer to participate any time during the pregnancy completing the first baseline questionnaire on general health and exposures before and during pregnancy. Further follow-up information is obtained with six additional online questionnaires completed by the mothers at 6 and 18 months after delivery and when children turn 4, 7, 10 and 13 years of age. The Ethical Committee of the San Giovanni Battista Hospital and CTO/CRF/Maria Adelaide Hospital of Turin approved the NINFEA study (approval N. 0048362, and subsequent amendments) and all the participants gave informed consent at enrolment.

We used the NINFEA database version 02.2019 that consists of 6625 mothers and 7423 pregnancies. Data on demographic and socioeconomic factors of the family are collected with the baseline questionnaire completed during pregnancy. Using this data (namely parental age, cohabitation status, education, country of birth and occupation, house size and type and family size) and the external data from the Italian 2011 EUSILC survey, an indicator of the equivalised total disposable household income at baseline (the Equivalised Household Income Indicator (EHII)) has been derived for the NINFEA participants. Child weight and height data are collected at each follow-up questionnaire. Child’s birth weight, gestational age at birth and weight at 6 months of age were ascertained from the 6-month questionnaire while weights at 18 months of age were obtained from the 18-month questionnaire. Weight and height data at 4, 7 and 10 years of age were gathered from the corresponding follow-up, with 4232, 2152 and 973 children having complete data on both weight and height at 4-year, 7-year and 10-year respectively. These data were used to calculate child body mass index (BMI).

**Follow-up response rates**

For each follow-up questionnaire, we identify the subset of participants for whom at the date of the database download (October, 2019) the time of the questionnaire completion was expired (eligible population) and among them we define as respondent a child whose mother started the completion of the index questionnaire. The response rate is then calculated in the eligible as the ratio of number of respondents over the total number of participants. In particular completion of the questionnaires expires as follows:

- 6-month follow-up: more than 24 months from date of conception
- 18-month follow-up: more than 30 months from date of delivery, or if the latter is missing more than 36 months from date of conception
- 4-year follow-up: more than 59 months from date of delivery;
- 7-year follow-up: more than 96 months from date of delivery
- 10-year follow-up: more than 132 months from date of delivery
- 13-year follow-up: more than 168 months from date of delivery

Twins are followed-up until the 18-month questionnaire and therefore are not part of the eligible population for the 4-, 7-, 10- and 13-year follow-up.

**Table S1 Results**


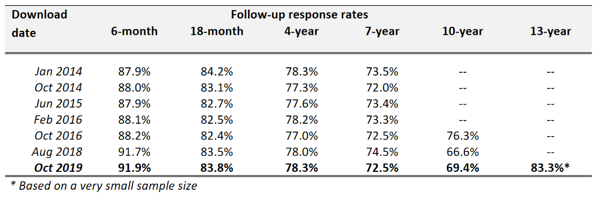

Supplement: Supplementary file 3 — Additional file 3. Additional information about the NINFEA cohort. [file 12916_2021_2155_MOESM3_ESM.docx]
